# The Impact of Different Cultivation Practices on Surface Runoff, Soil and Nutrient Losses in a Rotational System of Legume–Cereal and Sunflower

**DOI:** 10.3390/plants11243513

**Published:** 2022-12-14

**Authors:** Aikaterini Molla, Elpiniki Skoufogianni, Alexios Lolas, Konstantinos Skordas

**Affiliations:** 1 Laboratory of Soil Science, Department of Agriculture, Crop Production and Rural Environment, University of Thessaly, 38446 Volos, Greece; 2 Laboratory of Agronomy and Applied Crop Physiology, Department of Agriculture, Crop Production and Rural Environment, University of Thessaly, 38446 Volos, Greece; 3 Laboratory of Marine Biology, Department of Agriculture, Ichthyology and Aquatic Environment, University of Thessaly, 38446 Volos, Greece

**Keywords:** cropping system, rotation, tillage, phosphorus, potassium, natural rainfall, Greece

## Abstract

Soil erosion is among the biggest problems in the agricultural sector that can affect ecosystems and human societies. A field of 5° slope was selected to study the runoff, soil and nutrient loss as well as crop productivity in different treatments—conventional tillage (CT) vs. no-tillage (NT), plant vs. no plant cover, contour cultivation (CC) vs. perpendicular to the contour cultivation, (PC) under natural rainfall. The experiment was conducted in central Greece in two cultivation periods. In autumn, the field was cultivated with intercropping *Triticosecale* and *Pisum sativum* and in spring with sunflower. The total rainfall was 141.4 mm in the 1st year and 311 mm in the 2nd. We found that runoff in the treatment of no tillage with contour cultivation was 85% lower in both years compared to the no tillage-no plant control. Therefore, the contour cultivation-no tillage treatment had a positive effect by decreasing phosphorus and potassium loss from soil: indeed, there was a decrease in P and K by 55% and 62%, respectively, in the NT compared to the CC treatments. We conclude that the NT-CC treatment with plant cover was the most effective in reducing water runoff and soil nutrient loss and increasing yield.

## 1. Introduction

In recent years, the increased demand for food as a result of the increase in the global population has led to the exploitation of greater areas of agriculture [1]. Among the most important global problems in agricultural land use is soil erosion. It has been found that 80% of agricultural fields suffer from severe erosion impacts [2] (Pimentel and Burgess, 2013). Sloping lands cause more than the 60% of soil erosion [3]. The factors that can affect soil erosion can be divided into two categories: those occurring naturally and human induced. A number of studies have shown that slope gradient is the main natural factor that affects tillage erosion, and tillage erosion increases along with the increase in slope gradient [4,5,6]. Soil erosion in agriculture is mainly caused by rainfall (water-induced erosion), leading to land degradation, surface runoff, and soil and nutrient loss [7,8].

The human factors involved in the soil erosion process are farming practices and cropping systems. Proper tillage direction can affect runoff and soil and nutrient loss. Contour tillage is a more sustainable practice in comparison to that usually expected in flat fields (in straight lines) or along-the-slope tillage. Adverse effects become more pronounced under intensive rainfall events. Contour cultivation on fields with a high incline can decrease soil erodibility, thus increasing topsoil resistance [9]. Due to soil erosion, pollution by NPK borne onto eroded soil particles has become a major threat to surface waters. Globally, due to soil erosion, approximately 95% of phosphorus, 55% of nitrogen and up to 40% of carbon are being carried in rivers and deposited in their sediments [10]. In Europe, 12% of agricultural fields are negatively affected by erosion caused by water and this costs the EU-27 an approximately EUR 0.7–14.0 billion [11].

Soil erosion can cause ecological problems such as eutrophication of surface waters, lakes and reservoirs and can have severe negative impacts on the aquatic biota. Soil erosion can also lead to economical losses for farmers as well to a reduction in agricultural productivity [11,12,13].

In recent years, conservation tillage has been mentioned as an effective way to reduce soil erosion and, therefore, minimize soil and nutrient loss [14,15]. Conservation agriculture has three main principles—no-tillage cultivation, crop rotation and the use of permanent cover crops [16,17].

Regardless of the above reported advantages of conservation agriculture, especially for the Mediterranean countries, very often farmers and local communities believe that a field with continuous cover crops or intercropping, as well the use of minimum or no-tillage cultivation, is a “dirty” action [18,19].

Farmers should be taught the benefits of sustainable agricultural management, which is necessary to avoid soil and nutrient loss as well as to improve the physical and chemical characteristics of soil [14,19].

Greece, in the Mediterranean, is a country with a high risk of soil and nutrient loss due to soil erosion. This is due to the many sloping cultivated fields and the climate that is characterized by warm and rainy winters and erosive rains. Intensive rains in combination with hot and dry summers have intensified the soil erosion problem [19,20].

Although some studies have been conducted to evaluate the influence of soil tillage systems on surface runoff, soil and nutrient transport from agricultural fields [21,22,23] worldwide, not much information exists concerning Greece. Furthermore, studies that assess the effect of soil tillage (contour farming, CF, and non-CF) on the surface runoff are also rare.

Additionally, only a few studies have been conducted regarding the effects of a rotation system with legume–cereal and sunflower on runoff, soil, nutrient loss and plant biomass.

For that reason, the aim of this work was to study the effect of tillage (conventional and no tillage), planting direction (parallel and perpendicular to the contours), and vegetation cover (with or without crops of autumn and spring cultivations) on the runoff, soil loss, nutrient loss (recorded with Olsen P and exchangeable K) and plant total biomass.

## 2. Results

### 2.1. Meteorological Data

The meteorological data were recorded from an automatic station installed next to the experimental area.

**Figure 1:**
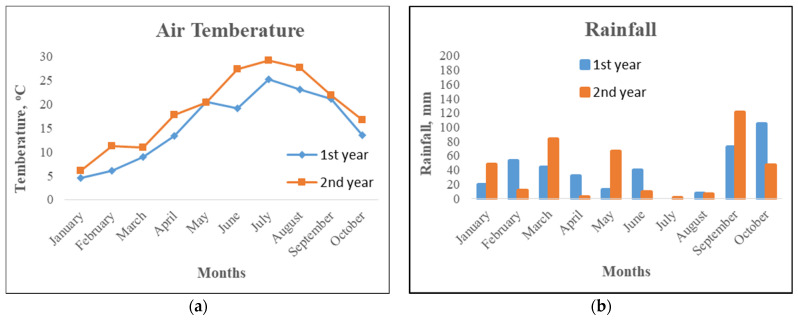
Average monthly air temperature (**a**) and total rainfall (**b**) occurring in the studied area during the growing periods (1st and 2nd growing years).

Air temperature was at least 2–3 °C higher during the 2nd year of the experiment in almost all months. The total precipitation from March to October was 314.9 and 340 mm in 2015 and 2016, respectively (Figure 1).

### 2.2. Soil Analyses

The soil was clay loam (38.41% sand, 36.11% clay, 25.48% silt), with a pH of 8.21 and organic matter content of 1.65%. The physicochemical properties of the soil are shown in Table 1.

### 2.3. Runoff Events Results

In total, 11 runoff events were conducted over the rainy season between the beginning of March and the end of May for the autumn cultivation and from mid-September to mid–October for the two experimental years. Specifically, three (March to May) and two (September to October) runoff events were measured in the 1st year, and three (March to May) and three (September to October) in the 2nd year. The rainfall, a characteristic from which runoff was generated, is shown in Table 2. The total amount of rainfall that resulted in runoff was 141.4 mm in 2015 and 310.9 mm in 2016, representing 45% and 91% of the total precipitation from March to October.

In order to evaluate the reduction in runoff, the RRB in % was calculated. The values of the RRB in % confirmed that the no-tillage treatments presented a decrease in runoff volumes in comparison to conventional blocks. In all four runoff events, no-tillage parallel to the contours caused a greater reduction than tillage perpendicular to the contours (Table 3).

The results of the runoff volumes are illustrated in Table 4. The runoff volumes of all treatments were lower in comparison to the control plots (no-tillage and no plant); and in 10 out of the 11 runoff events, the difference was statistically significant. The runoff values, from lowest to highest, follow the order: TR1 < TR2 < TR4 < TR5 < TR3 < TR6 < control. The TR1 (no tillage-planting parallel to the contour direction-plant) plots had a lower runoff volume. The highest runoff was observed for tillage perpendicular to the contour. Additionally, greater runoff volumes were observed in NT (no-tillage) plots than in CT (conventional tillage), regardless of the cultivated soil direction (parallel or perpendicular to the contour). During the 1st year, the total rainfall was 141.4 mm and the runoff values ranged from 5.004 (TR1) to 13.396 m^3^ ha^−1^ (control), while during the 2nd year, the total rainfall was 310.9 mm and the runoff volumes ranged from 3.4112 (TR1) to 21.096 m^3^ ha^−1^ (control).

### 2.4. Soil Loss Results

The soil loss concentrations are reported in Table 5. In all six treatments (TR1, TR2, TR3, TR4, TR5, and TR6), soil loss was lower in comparison to the control (no-tillage and no plant); and in 10 out of the 11 runoff events, the difference was statistically significant (RE3, RE4, RE5, RE6, RE7, RE8, RE9, RE10, and RE11). The soil loss rates followed the order TR1 < TR2 < TR4 < TR5 < TR3 < TR6 < control. The TR1 plots had a statistically significant difference only in the RE9 runoff event (110.7 mm rainfall). Larger soil losses were generally measured in the plots in which the tillage was performed perpendicular in the contour. Furthermore, the NT (no-tillage) produced lower soil loss amounts in comparison to the CT (conventional tillage), regardless of the direction of cultivation (either parallel or perpendicular to the contours). During the 1st year, out of a total rainfall of 141.4 mm, the soil loss values ranged from 0.953 (TR1) to 12.325 m^3^ ha^−1^ (control). During the 2nd year, out of a total rainfall of 310.9 mm, the runoff volumes ranged from 2.3399 (TR1) to 43.691 m^3^ ha^−1^ (control). The different land treatments decreased the sediment loss by 71–92% in the 1st year and by 67–95% in the 2nd year. The measurement of the sediment reduction benefit (SRB in %) showed that no-tillage reduced soil loss to a greater amount in comparison to conventional tillage. The reduction in no-tillage parallel to the contour ranged from 15.7 to 60.3%, while reduction in tillage perpendicular to the contour ranged from 18 to 43.1% (Table 6).

### 2.5. Nutrient Loss Results

The concentrations of the K and P losses are presented in Table 7 and Table 8. According to the results, in all treatments, the potassium and phosphorus losses were lower in comparison to in the control plots (no-tillage and no plant). The reduced potassium values ranged from 39% (TR1) to 72% (TR6) in the 1st year and from 47% (TR1) to 89% (TR6) in the 2nd year for a total rainfall of 141.4 and 310.9 mm, respectively. In the case of phosphorus, the decrease ranged from 35% (TR1) to 86% (TR6) in the 1st year and from 40% (TR1) to 82% (TR6) in the 2nd year.

Comparing the direction of planting tillage (parallel and perpendicular to the contour), the concentrations of potassium and phosphorus losses were reduced in tillage parallel to the contour. Additionally, the decreases in potassium and phosphorus losses were lower in no-tillage plots in comparison to conventional tillage. Analyses of variances were used to compare the amount of potassium and phosphorus losses in the different treatments for the two cultivation years in which total precipitation during the studied periods (March to October) was 141.4 and 310.9 mm in the 1st and 2nd years, respectively. The results (Table 7 and Table 8) show that there is a significant difference between all the different treatments and the control plots.

### 2.6. Total Biomass Results

As shown in Table 9 and Table 10, during the 1st and 2nd years, the total biomass of the intercropping *Triticosecale–Pisum sativum* in no-tillage treatment, with tillage parallel to the contour was greater than the total biomass in the other three treatments. The NT-PPACD-P treatment had a statistically significant difference with the CT-PPECD-P plots, for both cultivated years. That treatment was higher by 17%, 25% and 33% in comparison to CT-PPACD-P, NT-PPECD-P, CT-PPECD-P during the 1st year and 18%, 26% and 31% during the 2nd year, respectively.

As illustrated in Table 11 and Table 12, during both cultivation years, the plots with no-tillage and tillage parallel to the contour (NT-PPACD-P) presented a higher total yield—5350 and 5970 kg ha^−1^—during the 1st and 2nd years, respectively. Statistically significant differences were observed between the NT-PPACD-P and CT-PPECD-P treatments.

Furthermore, during the 2nd year the total biomass was greater compared to the 1st year in both cultivations (intercropping *Triticosecale–Pisum sativum* and *Helianthus annuus*). The increase in total yield was probably attributed to the positive impact of the residues which were incorporated into the field after the harvest of the intercropping *Triticosecale–Pisum sativum*.

## 3. Discussion

In this research, 11 rainfall events were generated by natural precipitation during the two cultivation years. The runoff values according to the results were lower compared to the no tillage-planting parallel to the contour–with plant treatment. Specifically, in the 1st year, the runoff values ranged from 5.004 (TR1) to 13.396 m^3^ ha^−1^ (control), while during the 2nd year, the runoff volumes ranged from 3.4112 (TR1) to 21.096 m^3^ ha^−1^ (control). It has to be mentioned that in the 2nd year, precipitation was 55% higher compared to in the 1st cultivation year. Similar results were observed by other studies [9,24,25]. On the other hand, Kebede et al. [26] reported a lower reduction in runoff (12–39%), using alternative soil erosion amendments (Anionic polyacrylamide, gypsum, lime, and biochar) in comparison to the current investigation results (a reduction from 62 to 86%).

Soil loss results indicated that the different tillage practices decreased sediment loss by 71–92% in the 1st year and by 67–95% in the 2nd year. The lowest reduction was obtained by the no tillage-planting parallel to the contours–with plant treatment. Furthermore, the measurement of the sediment reduction benefit showed that no tillage provoked a higher reduction in soil loss compared to conventional practice. Berihun et al. [8] found that different land management practices (no crop cultivation on steep slopes > 30%, Khat plantation, forage production, reforestation on communal and hilly croplands) resulted in a reduction in soil loss by 32–95%. Comparing our results with other studies, it can be verified that NT cultivation in lands with slope can significantly reduce soil loss [9,17]. Kurothe et al. [21] found that the average soil loss in NT was 37.2% less than in CT. Additionally, Merten et al. [22] indicated a decrease in soil loss of more than 70% using no tillage cultivation. Additionally, tillage parallel to the contour is more effective in decreasing sediment loss [27].

Furthermore, no tillage-planting parallel to the contour had a positive effect on the decrease in potassium and phosphorus content. The same results are mentioned by Peri et al. [28]. It can be said that agricultural practices such as soil tillage play a significant role in nutrient loss [10]. Wolka et al. [29] mentioned that tillage management can affect the nutrient transfer by the surface runoff. According to the literature, there are no studies which have been conducted for the investigation of positive or negative impacts of conventional tillage and no-tillage in combination with tillage parallel and perpendicular to the contour cultivation to the reduction in exchangeable potassium and extractable phosphorus.

Tillage parallel to the contour presented higher total biomass in both cultivations. Specifically, the total yield in NT-PPACD-P was higher by 17%, 25% and 33% in comparison to CT-PPACD-P, NT-PPECD-P, CT-PPECD-P during the 1st year and 18%, 26% and 31% during the 2nd year for *Triticosecale–Pisum sativum* intercropping. In sunflower cultivation, the biomass was 5350 and 5970 kg ha^−1^ during the 1st and 2nd years, respectively. Our results are in agreement with other studies [30,31]. According to our results, intercropping legume–cereal and sunflower cultivation increased the biomass in the 2nd year of the experiments. That legume intercropping in a rotation system promotes an advantageous increase in crop production is also indicated by other studies [32,33].

## 4. Materials and Methods

### 4.1. Study Area

**Figure 2:**
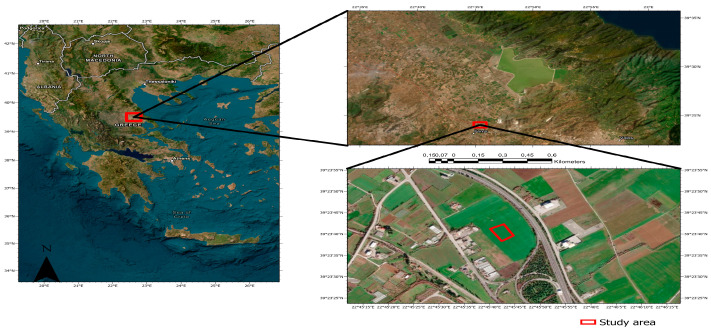
Location of the study area.

Thus, experiment was established in a field with a slope of at least 5% at the Experimental Station of the University of Thessaly (Larissa—Greece). The studied area, which has latitude of 39°37′30″ and a longitude of 22°22′51″, was located at an altitude of 80 m above sea level (Figure 2). The climate in the area is characterized as Mediterranean, with hot and dry summers as well as cold and wet winters.

### 4.2. Soil Analyses

A soil sample from the field was taken from a depth of 0–30 cm using a steel auger, before sowing. The soil sample was transported to the soil laboratory, air-dried and then sieved through a 2mm sieve. The pH (1:2.5 d. H_2_O) of the soil was determined along with its electrical conductivity (1:5 d. H_2_O),concentration of calcium carbonate (CaCO_3_) using a calcimeter, percentage (%) of sand, clay and silt using the Bouyoukos method, organic matter using the Walkley–Black method, total nitrogen (Kjeldahl method), available soil P (Olsen method, analyzed with ammonium vanadomolybdate/ascorbic blue and measured in a UV spectrophotometer at 882 nm) and exchangeable Κ (1:10 at 1M CH_3_COONH_4_ pH 7, analyzed in a flame photometer). All the analyses were carried out according to Rowell (1994) [34].

### 4.3. Field Experiment

The experiment included various combinations of cultivation treatments (conventional tillage and no-tillage), different cultivated soil direction (parallel and perpendicular to the contours), and vegetation covers (with and without crops), resulting in 7 treatments with three replicates each (treatments are explained in Table 13). The plots were 132 m^2^ in size (6 m in width × 22 m in length). A split-plot experimental design was implemented.

The experiment was conducted in two cultivated years. During the experiments, all the necessary cultivation practices were conducted. Conventional tillage included ploughing to a depth of approximately 25 cm in both autumn and spring. For the autumn cultivation, tillage took place on the 6 December in the 1st year and on the 8 November in the 2nd year. For the spring, crop tillage was carried out on the 30 June 2015 and on the 12 June 2016.

All the plots were sprayed with herbicide glyphosate (at 5 L/ha) at least one month before the autumn cultivation in the 1st year of the experiment. Additionally, during the autumn cultivation, the no-tillage plots were sprayed using herbicide glyphosate (3 L/ha) in late March, during both cultivation years.

The plots were sown with intercropping *Pisum sativum* (140 kg ha^−1^) and *Triticosecale* (60 kg ha^−1^) in the autumn period and with *Helianthus annuus* (85.000 seeds ha^−1^) in the spring period.

For the two cultivation periods, the following crop sequence was used for the experiments: (a) winter rotation of legume–cereal (2014/2015); (b) summer sunflower (2015); (c) winter rotation of legume–cereal (2015/2016); (d) summer sunflower (2016).

During the autumn cultivation, N was applied as basic (1/3 at sowing) and as top-dressing fertilizer (2/3 at the end of March). Phosphorus (270 kg P_2_O_5_ ha^−1^) and K (270 kg K_2_O ha^−1^) were applied at the same time with sowing. During the spring cultivation, the blocks were treated with N (40 kg N ha^−1^), P (60 kg P_2_O_5_ ha^−1^) and K (60 kg K_2_O ha^−1^) during sowing.

For the 1st year, the autumn cultivation was harvested on the 5 June and the *Helianthus annuus* plants on the 17 October. For the 2nd year, the harvest was performed on the 3 June for the intercropping cultivation *Pisum sativum* and *Triticosecale* and on the 16 October for the *Helianthus annuus*. The harvest of the plots with plants was conducted using a frame of 1 m^2^. The frame was placed in 4 random places within each plot and the total biomass from inside the frame was collected and harvested at a height of 1 cm above soil level. In the case of the intercropping cultivation of *Pisum sativum* and *Triticosecale*, the two crops were separated. Additionally, the plants of *Pisum sativum* were separated into stems, seeds and pods and the *Triticosecale* plants into to stems and spikes. After the harvest of the autumn cultivation, the crop residues of the intercropping *Triticosecale* and *Pisum sativum* were incorporated into the field.

### 4.4. Measurement of Runoff, Soil and Nutrient Loss

This study was conducted under natural rainfall conditions. Every plot was enclosed by a metal pipes system, so that the runoff was discharged into large containers which were installed into the ground at the down slope edge of each plot. In each container, a plastic bag was used; and after a significant natural rainfall event, the bags were put in boxes and transported to the laboratory, where they were left to settle until the sediment subsided. Then, the runoff volume from each box was collected and weighed. The sediment samples were gathered and dried at 60 °C for 48 h. From these samples, soil loss, the Olsen P (extraction at 1:20 with 0.5 M sodium bicarbonate (NaHCO_3_) and the exchangeable K (extracted at 1:5 with 1 M CH_3_CHOONH_4_) were measured (methods according to Rowel 1994).

In order to evaluate the way that the different tillage treatments affect the runoff and soil loss, two indices were chosen: (a) runoff reduction benefit (RRB) in % and (b) sediment reduction benefit (SRB) in % [9].

These indices were calculated using the following equations:

If (RCT − RNT) > 0 then RRB = ((RCT − RNT)/RCT) × 100(1)

If (RCT − RNT) < 0 then RRB = ((RCT − RNT)/RNT) × 100(2)

If (SCT − SNT) > 0 then SRB = ((SCT − SNT)/SCT) × 100(3)

If (SCT − SNT) < 0 then SRB = ((SCT − SNT)/SNT) × 100(4)

where

RCT is the runoff volume (m^3^) in the conventional tillage blocks,

RNT is the runoff volume (m^3^) in the no-tillage blocks,

SCT is soil loss (kg/ha) in the conventional tillage blocks, and

SNT is soil loss (kg/ha) in the no-tillage blocks.

### 4.5. Statistical Analysis

The data were analyzed using the Statgraphics plus 8.1 statistical analysis software for the analysis of variance at the 95% significance level (*p* < 0.05) and the LSD test was employed as a means of indicating the significance of differences among treatments.

## 5. Conclusions

In this study, we evaluated the impacts of no-tillage on runoff, soil and nutrient losses under natural rainfall in comparison to conventional agriculture. In addition, we investigated the effect of planting direction (parallel and perpendicular to the contour).

The results showed that the runoff volumes, the soil and nutrient losses were generally higher in CT than in NT, regardless of the cultivated soil direction. In the case of tillage direction, tillage parallel to the contour had a positive impact on the investigated parameters (runoff, soil and nutrient losses).

Furthermore, The RRB and SRB values confirm that no-tillage parallel to the contour caused a greater reduction than that in tillage perpendicular to the contour in runoff and in soil loss.

Since potassium and phosphorus nutrients (K and P) are necessary for plant growth, their losses, due to runoff, can lead to a detrimental impact on yield production, especially when the fertilizers are expensive. In the current study, significant differences have been observed regarding potassium and phosphorus losses between the different treatments. Specifically, the decrease was higher in plots cultivated parallel to the contour and with no tillage.

Additionally, plant biomass yield was affected by tillage direction. No-tillage planting parallel to the contour had a positive impact on crop production in comparison to the other treatments. Specifically, intercropping *Triticosecale–Pisum sativum* and *Helianthus annuus* yield was higher in the NT-PPACD-P plots in comparison to CT-PPACD-P, NT-PPECD-P, CT-PPECD-P during the 1st and 2nd years. Additionally, it should be noted that during the 2nd year, plant biomass was greater than that of the 1st year. This probably means that the residues that remained in the field after the 1st year harvest positively influenced production in the 2nd year.

To sum up, for Greece’s climate, the best agriculture management for sloping fields is for them to be cultivated using no tillage and planting should be conducted parallel to the contour. Finally, the cultivated plant system legume–cereal and sunflower is a promising crop rotational process in the reduction in soil and nutrient losses.

## Figures and Tables

**Table 1:** Physicochemical properties of the used soil.

Physicochemical Properties	Value
pH *	8.21
E.C. * (μS cm^−1^)	435
CaCO_3_ * (%)	16.5
Organic matter (%)	1.65
Total nitrogen (%)	0.08
Olsen phosphorus (P) (mg kg^−1^)	21.24
Exchangeable Potassium (Κ) (mg kg^−1^)	216.06
Sand (%)	38.41
Clay (%)	36.11
Silt (%)	25.48

* pH: Hydrogen Potenz; E.C.: Electrical Conductivity; CaCO_3_: calcium carbonate.

**Table 2:** Characteristics of the rainfall events generating runoff volumes.

Runoff Event	Days of Rain	Sampling Day	Rainfall Amount (mm)	Runoff Event	Days of Rain	Sampling Day	Rainfall Amount (mm)
1st year				2nd year			
Intercropping *Triticosecale–Pisum sativum* cultivation (2014/2015)				Intercropping *Triticosecale–Pisum sativum* cultivation (2015/2016)			
RE1	19/3/15–31/3/15	1/4/15	44.2	RE6	7/3/16–16/3/16	17/3/16	68.9
RE2	1/4/15–4/5/15	5/5/15	31.6	RE7	18/3/16–31/4/16	1/5/16	16
RE3	6/5/15–18/5/15	19/5/15	12.6	RE8	2/5/16–1/6/16	2/6/16	66
	Total		88.4		Total		150.9
*Helianthus annuus* (2015)				*Helianthus annuus* (2016)			
RE4	1/9/15–31/10/15	1/10/15	35.8	RE9	1/9/16–12/9/16	13/9/16	110.7
RE5	2/10/15–8/10/15	9/10/15	17.2	RE10	14/9/16–24/9/16	25/9/16	20
				RE11	26/9/16–15/10/16	14/10/16	29.3
	Total		53		Total		160

**Table 3:** Runoff reduction benefit (RRB) over the four cultivation periods.

		Runoff Reduction Benefit (RRB) in %	
Cultivation Period	Runoff Event	Tillage Parallel to Contour	Tillage Perpendicular to Contour
winter rotation of legume–cereal (2014/2015)	88.4 mm	2.0	1.8
summer sunflower (2015)	53 mm	29.3	0.6
winter rotation of legume–cereal (2015/2016)	150.9 mm	13.4	12.3
summer sunflower (2016)	160 mm	15.3	12.7

**Table 4:** Mean values of runoff volumes (m^3^ ha^−1^) in the seven treatments of the two cultivation years.

Runoff Event	Rainfall Amount (mm)	Runoff (m^3^ ha)							
		Control	TR1	TR2	TR3	TR4	TR5	TR6	LSD
1st year									
RE1	44.2	1.6040 ^b^	1.2776 ^a^	1.3086 ^ab^	1.514 6 ^ab^	1.4441 ^ab^	1.4639 ^ab^	1.5381 ^ab^	0.09969
RE2	31.6	2.0535 ^c^	1.8167 ^a^	1.8453 ^ab^	2.0209 ^c^	1.9285 ^abc^	1.9849 ^bc^	2.0345 ^c^	0.05032
RE3	12.6	0.7255 ^c^	0.5582 ^a^	0.5702 ^a^	0.6533 ^ab^	0.6414 ^ab^	0.6402 ^ab^	0.6745 ^ab^	0.04357
Total 1 (RE1, RE2, RE3)	88.4	4.383 ^d^	3.6503 ^a^	3.7241 ^ab^	4.1888 ^cd^	4.0140 ^abc^	4.0890 ^bcd^	4.2471 ^cd^	0.12064
RE4	35.8	2.8133 ^b^	0.3514 ^a^	0.4059 ^a^	0.5488 ^a^	0.4585 ^a^	0.5230 ^a^	0.6890 ^a^	0.17033
RE5	17.2	6.2000 ^d^	1.0027 ^a^	1.5089 ^ab^	2.0018 ^bc^	1.8643 ^b^	1.8138 ^b^	2.7221 ^c^	0.26517
Total 2 (RE4, RE5)	53	9.0133 ^d^	1.3540 ^a^	1.9148 ^ab^	2.5506 ^cd^	2.3228 ^ab^	2.3368 ^ab^	3.4111 ^c^	0.32597
2nd year									
RE6	68.9	2.0503 ^d^	0.6763 ^a^	0.7813 ^ab^	1.0563 ^bc^	0.8823 ^ab^	1.0065 ^b^	1.3260 ^c^	0.09997
RE7	16	1.0400 ^c^	0.1570 ^a^	0.1814 ^ab^	0.2453 ^ab^	0.2049 ^ab^	0.2337 ^ab^	0.3079 ^b^	0.04255
RE8	66	4.4460 ^c^	0.6478 ^a^	0.7484 ^a^	1.0118 ^ab^	0.8452 ^a^	0.9642 ^ab^	1.3517 ^b^	0.16488
Total 3 (RE6, RE7, RE8)	150.9	7.5363 ^d^	1.4811 ^a^	1.7110 ^ab^	2.3133 ^bc^	1.9324 ^ab^	2.2044 ^b^	2.9857 ^c^	0.23789
RE9	110.7	7.0743 ^d^	1.0865 ^a^	1.2552 ^ab^	1.6971 ^bc^	1.4176 ^ab^	1.6171 ^abc^	2.1305 ^c^	0.19230
RE10	20	1.2610 ^c^	0.1963 ^a^	0.2268 ^a^	0.3066 ^ab^	0.2561 ^a^	0.2922 ^ab^	0.3849 ^b^	0.03651
RE11	29.3	5.2243 ^c^	0.6478 ^a^	0.7484 ^ab^	1.0118 ^ab^	0.8452 ^ab^	0.9642 ^ab^	1.270 ^b^	0.18231
Total 4 (RE9, RE10, RE11)	160	13.560 ^c^	1.9306 ^a^	2.2304 ^a^	3.0155 ^ab^	2.5190 ^a^	2.8735 ^ab^	3.7857 ^b^	0.40142

Different letters within each line indicate statistically significant differences between the treatments at the *p* < 0.05 level.

**Table 5:** Mean values of soil loss (kg ha^−1^) volumes in the seven treatments of the two cultivation years.

Runoff Event	Rainfall Amount (mm)	Soil Loss (kg ha^−1^)							
		Control	TR1	TR2	TR3	TR4	TR5	TR6	LSD
1st year									
RE1	44.2	2.504 ^d^	0.311 ^a^	0.323 ^a^	0.699 ^bc^	0.367 ^a^	0.475 ^ab^	0.867 ^c^	0.08552
RE2	31.6	0.723 ^c^	0.256 ^a^	0.350 ^ab^	0.463 ^b^	0.353 ^ab^	0.413 ^ab^	0.644 ^c^	0.05817
RE3	12.6	0.563 ^d^	0.057 ^a^	0.068 ^a^	0.141 ^b^	0.125 ^ab^	0.144 ^b^	0.232 ^c^	0.02403
Total 1 (RE1, RE2, RE3)	88.4	3.789 ^e^	0.624 ^a^	0.741 ^ab^	1.303 ^c^	0.8456 ^ab^	1.0312 ^bc^	1.7440 ^d^	0.11603
RE4	35.8	5.283 ^e^	0.269 ^a^	0.592 ^ab^	1.287 ^c^	0.726 ^b^	1.183 ^cd^	1.647 ^d^	0.14450
RE5	17.2	3.252 ^b^	0.059 ^a^	0.095 ^a^	0.126 ^a^	0.102 ^a^	0.120 ^a^	0.194 ^a^	0.10938
Total 2 (RE4, RE5)	53	8.535 ^d^	0.328 ^a^	0.686 ^a^	1.412 ^cd^	0.828 ^ab^	1.303 ^bc^	1.841 ^c^	0.16696
Total 1, 2	141.4	12.32	0.95	1.43	2.72	1.67	2.33	3.59	
2nd year									
RE6	68.9	10.070 ^e^	0.519 ^a^	1.319 ^ab^	2.277 ^c^	1.397 ^b^	2.477 ^cd^	3.171 ^c^	0.27725
RE7	16	2.331 ^e^	0.120 ^a^	0.284 ^ab^	0.575 ^cd^	0.325 ^b^	0.529 ^c^	0.736 ^d^	0.06338
RE8	66	7.046 ^b^	0.497 ^a^	1.091 ^a^	2.181 ^a^	1.338 ^a^	2.372 ^a^	3.037 ^a^	1.14313
Total 3 (RE6, RE7, RE8)	150.9	19.447 ^d^	1.136 ^a^	2.394 ^ab^	5.034 ^bc^	3.06 ^ab^	5.378 ^bc^	6.944 ^c^	1.12578
RE9	110.7	16.137 ^e^	0.833 ^a^	2.187 ^b^	3.889 ^c^	2.245 ^c^	3.979 ^cd^	5.094 ^d^	0.39169
RE10	20	3.263 ^d^	0.151 ^a^	0.364 ^ab^	0.736 ^c^	0.406 ^b^	0.719 ^ac^	0.920 ^c^	0.07830
RE11	29.3	4.844 ^e^	0.221 ^a^	0.484 ^ab^	1.053 ^cd^	0.728 ^bc^	0.923 ^bcd^	1.260 ^d^	0.16329
Total 4 (RE9, RE10, RE11)	160	24.244 ^d^	1.204 ^a^	3.035 ^b^	5.677 ^c^	3.379 ^b^	5.621 ^c^	7.275 ^c^	0.59079
Total 3, 4	310.9	43.69	2.34	5.43	10.71	6.44	11.00	14.22	

Different letters within each line indicate statistically significant differences between the treatments at the *p* < 0.05 level.

**Table 6:** Sediment reduction benefit (SRB) for the four cultivation periods.

		Sediment Reduction Benefit (RRB) in %	
Cultivation Period	Runoff Event	Tillage Parallel to Contour	Tillage Perpendicular to Contour
winter rotation of legume–cereal (2014/2015)	88.4 mm	15.7	18.0
summer sunflower (2015)	53 mm	52.1	36.4
winter rotation of legume–cereal (2015/2016)	150.9 mm	57.8	43.1
summer sunflower (2016)	160 mm	60.3	39.9

**Table 7:** Mean values of potassium loss (mg kg^−1^ soil) in the seven treatments of the two cultivation years.

Runoff Event	Rainfall Amount (mm)	K (mg kg^−1^ Soil)							
		Control	TR1	TR2	TR3	TR4	TR5	TR6	LSD
1st year									
RE1	44.2	0.819 ^f^	0.254 ^a^	0.257 ^a^	0.433 ^d^	0.304 ^b^	0.390 ^c^	0.546 ^e^	0.003425
RE2	31.6	0.624 ^f^	0.195 ^a^	0.246 ^b^	0.304 ^d^	0.281 ^c^	0.289 ^c^	0.340 e	0.003462
RE3	12.6	0347 ^e^	0.153 ^a^	0.179 ^b^	0.251 ^d^	0.195 ^c^	0.250 ^d^	0.261 ^d^	0.004039
Total 1 (RE1, RE2, RE3)	88.4	1.790 ^g^	0.602 ^a^	0.682 ^b^	0.987 ^e^	0.780 ^c^	0.930 ^d^	1.147 ^f^	0.005863
RE4	35.8	0.661 ^e^	0.244 ^a^	0.378 ^b^	0.507 ^cd^	0.438 ^bc^	0.478 ^c^	0.585 ^de^	0.029260
RE5	17.2	0.787 ^e^	0.353 ^a^	0.410 ^b^	0.585 ^d^	0.414 ^b^	0.476 ^c^	0.598 ^d^	0.004537
Total 2 (RE4, RE5)	53	1.448 ^e^	0.658 ^a^	0.731 ^a^	1.091 ^d^	0.848 ^b^	0.954 ^c^	1.184 ^d^	0.030488
Total 1, 2	141.4	3.24	1.26	1.41	2.08	1.63	1.88	2.33	
2nd year									
RE6	68.9	1.273 ^f^	0.394 ^a^	0.405 ^a^	0.844 ^d^	0.477 ^b^	0.608 ^c^	1.140 ^e^	0.005334
RE7	16	0.319 ^f^	0.099 ^a^	0.124 ^b^	0.172 ^d^	0.145 ^c^	0.146 ^c^	0.189 ^e^	0.002560
RE8	66	1.815 ^e^	0.935 ^a^	1.028 ^b^	1.315 ^d^	1.123 ^c^	1.309 ^d^	1.354 ^d^	0.018415
Total 3 (RE6, RE7, RE8)	150.9	3.407 ^g^	1.428 ^a^	1.557 ^b^	2.330 ^e^	1.745 ^c^	2.063 ^d^	2.683 ^f^	0.022885
RE9	110.7	2.511 ^e^	1.164 ^a^	1.353 ^b^	1.826 ^c^	1.482 ^b^	1.997 ^c^	2.193 ^d^	0.005711
RE10	20	0.674 ^e^	0.407 ^a^	0.413 ^a^	0.696 ^d^	0.471 ^b^	0.551 ^c^	0.703 ^e^	0.005952
RE11	29.3	0.989 ^e^	0.577 ^a^	0.597 ^a^	0.956 ^d^	0.668 ^b^	0.778 ^c^	0.976 ^de^	0.006863
Total 4 (RE9, RE10, RE11)	160	4.174 ^f^	2.147 ^a^	2.364 ^b^	3.478 ^d^	2.622 ^c^	3.326 ^d^	3.872 ^e^	0.061679
Total 3, 4	310.9	7.58	3.58	3.92	5.81	4.37	5.39	6.74	

Different letters within each line indicate statistically significant differences between the treatments at the *p* < 0.05 level.

**Table 8:** Mean values of phosphorus loss (mg kg^−1^ soil) in the seven treatments of the two cultivation years.

Runoff Event	Rainfall Amount (mm)	P (mg kg^−1^ Soil)							
		Control	TR1	TR2	TR3	TR4	TR5	TR6	LSD
1st year									
RE1	44.2	0.260 ^e^	0.103 ^a^	0.184 ^b^	0.222 ^cd^	0.186 ^b^	0.214 ^c^	0.225 ^d^	0.002800
RE2	31.6	0.396 ^e^	0.130 ^a^	0.161 ^b^	0.188 ^c^	0.164 ^b^	0.196 ^c^	0.366 ^d^	0.003956
RE3	12.6	0.301 ^f^	0.086 ^a^	0.120 ^b^	0.211 ^d^	0.160 ^c^	0.163 ^c^	0.244 ^e^	0.003644
Total 1 (RE1, RE2, RE3)	88.4	0.957 ^g^	0.319 ^a^	0.465 ^b^	0.629 ^e^	0.510 ^c^	0.565 ^d^	0.835 ^f^	0.006313
RE4	35.8	0.092 ^c^	0.044 ^a^	0.049 ^a^	0.065 ^b^	0.058 ^b^	0.063 ^b^	0.088 ^c^	0.002523
RE5	17.2	0.103 ^d^	0.027 ^a^	0.042 ^b^	0.069 ^c^	0.048 ^b^	0.067 ^c^	0.071 ^c^	0.003850
Total 2 (RE4, RE5)	53	0.195 ^f^	0.085 ^a^	0.087 ^b^	0.130 ^d^	0.113 ^c^	0.121 ^cd^	0.156 ^e^	0.004596
Total 1, 2	141.4	1.15	0.40	0.55	0.76	0.62	0.69	0.99	
2nd year									
RE6	68.9	0.402 ^e^	0.159 ^a^	0.249 ^b^	0.334 ^d^	0.284 ^c^	0.287 ^c^	0.345 ^d^	0.004214
RE7	16	0.201 ^e^	0.066 ^a^	0.082 ^b^	0.099 ^c^	0.083 ^b^	0.095 ^c^	0.184 ^d^	0.001881
RE8	66	1.571 ^f^	0.621 ^a^	0.833 ^b^	1.066 ^c^	0.837 ^b^	1.010 ^d^	1.272 ^e^	0.017213
Total 3 (RE6, RE7, RE8)	150.9	2.173 ^f^	0.846 ^a^	1.164 ^b^	1.499 ^d^	1.204 ^b^	1.392 ^c^	1.801 ^e^	0.018579
RE9	110.7	0.280 ^d^	0.135 ^a^	0.145 ^a^	0.203 ^c^	0.174 ^b^	0.191 ^bc^	0.205 ^c^	0.006617
RE10	20	0.083 ^c^	0.030 ^a^	0.036 ^a^	0.063 ^b^	0.056 ^b^	0.058 ^b^	0.080 ^c^	0.003761
RE11	29.3	0.079 ^c^	0.027 ^a^	0031 ^a^	0.049 ^b^	0.042 ^b^	0.045 ^b^	0.071 ^c^	0.003343
Total 4 (RE9, RE10, RE11)	160	0.443 ^e^	0.192 ^a^	0.212 ^a^	0.314 ^c^	0.271 ^b^	0.294 ^bc^	0.357 ^d^	0.008032
Total 3, 4	310.9	2.62	1.04	1.38	1.81	1.48	1.69	2.16	

Different letters within each line indicate statistically significant differences between the treatments at the *p* < 0.05 level.

**Table 9:** Biomass of the intercropping *Triticosecale–Pisum sativum* cultivation (kg ha^−1^) under different soil practices during the 1st year.

Yield, kg ha^−1^			CV %
Treatment	*Triticosecale-Pisum sativum*		
NT-PPACD-P	3034	b	16.4
CT-PPACD-P	2508	ab	8.3
NT-PPECD-P	2275	ab	23.6
CT-PPECD-P	2018	a	19.7
LSD		251.5	

Different letters at each column denote a statistically significant difference in the means according to the LSD test at the 95% significance level (*p* < 0.05). NT-PPACD-P: no tillage-planting parallel to the contour direction-plant. CT-PPACD-P: conventional tillage-planting parallel to the contour direction–plant. NT-PPECD-P: no tillage-planting perpendicular to the contour direction-plant. CT-PPECD-P: conventional tillage-planting perpendicular to the contour direction-plant.

**Table 10:** Biomass of the intercropping *Triticosecale–Pisum sativum* cultivation (kg ha^−1^) under different soil practices during the 2nd year.

Yield, kg ha^−1^			CV %
Treatment	*Triticosecale–Pisum sativum*		
NT-PPACD-P	3239	b	15.4
CT-PPACD-P	2646	ab	9.1
NT-PPECD-P	2412	a	12.6
CT-PPECD-P	2226	a	4.4
LSD		184.61	

Different letters at each column denote a statistically significant difference in the means according to the LSD test at the 95% significance level (*p* < 0.05). NT-PPACD-P: no tillage-planting parallel to the contour direction-plant. CT-PPACD-P: conventional tillage-planting parallel to the contour direction-plant. NT-PPECD-P: no tillage-planting perpendicular to the contour direction-plant. CT-PPECD-P: conventional tillage-planting perpendicular to the contour direction-plant.

**Table 11:** Biomass of the *Helianthus annuus* cultivation (kg ha^−1^) under different soil practices during the 1st year.

	Yield, kg ha^−1^		CV %
Treatment	*Helianthus annuus*		
NT-PPACD-P	5350	b	23.4
CT-PPACD-P	5230	b	10.5
NT-PPECD-P	4933	ab	5.1
CT-PPECD-P	3750	a	4.0
LSD		403.03	

Different letters at each column denote a statistically significant difference in the means according to the LSD test at the 95% significance level (*p* < 0.05). NT-PPACD-P: no tillage-planting parallel to the contour direction-plant. CT-PPACD-P: conventional tillage-planting parallel to the contour direction-plant. NT-PPECD-P: no tillage-planting perpendicular to the contour direction-plant. CT-PPECD-P: conventional tillage-planting perpendicular to the contour direction-plant.

**Table 12:** Biomass of the *Helianthus annuus* cultivation (kg ha^−1^) under different soil practices during the 2nd year.

	Yield, kg/ha		CV %
Treatment	*Helianthus annuus*		
NT-PPACD-P	5970	b	12.0
CT-PPACD-P	5337	ab	9.3
NT-PPECD-P	5037	ab	3.6
CT-PPECD-P	4597	a	18.7
LSD		356.92	

Different letters at each column denote a statistically significant difference in the means according to the LSD test at the 95% significance level (*p* < 0.05). NT-PPACD-P: no tillage-planting parallel to the contour direction-plant. CT-PPACD-P: conventional tillage-planting parallel to the contour direction–plant. NT-PPECD-P: no tillage-planting perpendicular to the contour direction-plant. CT-PPECD-P: conventional tillage-planting perpendicular to the contour direction-plant.

**Table 13:** Abbreviations and description of the treatments.

Treatments	Abbreviation	Treatment Description
Control	NT-WP	no tillage—without plant
TR1	NT-PPACD-P	no tillage-planting parallel to the contours—with plant
TR2	CT-PPACD-P	conventional tillage-planting parallel to the contours—with plant
TR3	CT-PACD-WP	conventional tillage-planting parallel to the contours—without plant
TR4	NT-PPECD-P	no tillage-planting perpendicular to the contours—with plant
TR5	CT-PPECD-P	conventional tillage-planting perpendicular to the contours—with plant
TR6	CT-PECD-WP	conventional tillage perpendicular to the contours—without plant

## Data Availability

Not applicable.
